# Annotations

**Published:** 1887-06-11

**Authors:** 


### ANNOTATIONS.

**Co-operation among the Working Classes.**

THE friends of co-operation have recently celebrated a Jubilee of their own. They have been both glad and sad: glad that something has been done; sad that that something should have been so small. Co-operation and isolation are two principles as opposite as the Poles. Co-operation is one of the highest developments of intelligence: isolation is what man has in common with the lion and the tiger, the boa and the crocodile. Isolation has had a long innings: co-operation has but recently taken the bat in hand. It is true the Founder of Christianity taught men the value and beauty of co-operation—as for example, "One is your master, even Christ, and all ye are brethren"; and also, "If any man will be great among you let him be your servant." The task of mankind is a task common to all mankind. It is the great task of subduing the earth to the service of men—a task which, according to the book of Genesis, was imposed upon the first historic man—"Be fruitful, and multiply and replenish the earth, and subdue it," said Jehovah. The question for consideration is this—given a common task, and given a large number of men for the performance of it, will those men accomplish the task better and more promptly by working everyone after his own pleasure, without general plan and purpose, or by all working in concert, each taking that part for which he is best fitted, and all labouring unitedly for the one great end? To ask the question is to answer it; and, speaking generally, it is certain that co-operation is as much better than isolation, as rule of three is better than cutting simple sums in arithmetic on a soft stick. Just now our object is to repeat what we have often urged before, that there are too many separate interests in the hospital world for that world to be either thoroughly efficient or thoroughly economical. The wiser and more conscientious among hospital managers should take this subject seriously to heart. The foolish and selfish will never do so. In union and concert all kinds of efficiency and success may be looked for. In isolation nothing may be expected but feebleness, discontent, and movement in a backward direction.

**A New Cure for Consumption.**

An evening contemporary reports that "considerable sensation has been created in medical circles in Vienna by the discovery of a supposed cure for consumption and other tubercular affections of the lungs or other parts of the body. The discoverer is Dr. Kolischer, a young operator in the clinical department of Professor Albert. Dr. Kolischer, starting on the assumption that tuberculosis occasionally heals naturally owing to the tubercles becoming calcined, hit upon the idea of causing artificial calcination by means of hypodermic injections of a compound described as *calcium phosphoricum*, into the limbs of persons affected with local tuberculosis. He made a number of experiments with a view to testing the merits of his discovery, and in each case the experiments turned out successful. At the last meeting of the Vienna Society of Physicians Dr. Kolischer read a paper on the results of his experiments, and introduced to the meeting several persons who had been cured by his method." That sounds very plausible, and to the uninitiated very conclusive. It is quite true that there is sometimes found in the lungs of deceased persons calcined or calcareous matter which is a degenerated state of tubercular disease; but how far such a degenerated condition of large tracts of lung-tissue may properly be called a "cure," is another question. However, abstract reasoning and weighing of pros and cons on such a subject is as profitless and futile as attempting to square the circle or to originate perpetual motion. Here is a test which it requires no medical knowledge to understand and apply. The greatest blockhead in the school may go to Dr. Kolischer and say: "You profess to cure consumption. There are hundreds of persons in various parts of the world who are manifestly dying of consumption. If your method be really a 'cure' there can be no difficulty in raising by subscription a sufficient sum to carry you about from town to town and from country to country curing and teaching until all but the very worst consumptives are restored to health. If you do this you will prove to all the world the reality of your cure, and you will become as famous as Moses or Goethe, or the illustrious Kaiser Wilhelm himself." Dr. Kolischer is presumably a humane man, he has found a cure which is easy and inexpensive; then let him at one and the same time gratify his humane instincts, fill his coffers with gold, and immortalise his name. All these things he will do without the least difficulty, provided his "cure" be real and not spurious. "The proof of a medical, as of any other pudding, is in the eating."

**The London Temperance Hospital.**

The London Temperance Hospital is an instance of a successful experiment. It was established fourteen years ago, with a view to demonstrating that sick people in the vast majority of cases do not require alcohol in any shape or form to effect a cure. Since its opening, 41,160 patients have received in-treatment, the rate of mortality during the whole of this period being only 5.8 per cent.; though, of course, the opening of the new casualty ward during the year, admitting many very serious accidents, has naturally somewhat raised this figure. An average of about 50 per cent. of the patients admitted are non-abstainers, and some of these leave the institution the friends of temperance, so that the hospital is well entitled to be considered as a factor in the progress of reform in this direction. Although the hospital has 120 beds, a half of them are kept vacant, for there is an existing debt of £6,500, and until this is cleared it is not considered prudent to extend present liabilities.

